# Misdiagnosis of constrictive pericarditis presenting with haemorrhagic pericardial effusion: a case report

**DOI:** 10.1093/ehjcr/ytz064

**Published:** 2019-05-10

**Authors:** Guoliang Li, Peng Liu, Dan Li, Yang Yan

**Affiliations:** 1 Arrhythmia Unit, Department of Cardiovascular Medicine, First Affiliated Hospital of Xi’an Jiaotong University, No. 277 Yanta West Road, Xi’an, P.R. China; 2 Department of Respiratory and Critical Care Medicine, First Affiliated Hospital of Xi’an Jiaotong University, No. 277 Yanta West Road, Xi’an, P.R. China; 3 Department of Cardiovascular Surgery, First Affiliated Hospital of Xi’an Jiaotong University, No. 277 Yanta West Road, Xi’an, P.R. China

**Keywords:** Constrictive pericarditis, Right heart failure, Pericardectomy, Case report

## Abstract

**Background:**

The symptoms and signs of constrictive pericarditis (CP) are often elusive at onset, with a long symptom-free period that may take weeks to decades to develop after an episode of CP or pericardial injury, leading to a misdiagnosis.

**Case summary:**

In this case, a 58-year-old man complained of lower extremity fatigue, intermittent chest tightness, and shortness of breath. He was first misdiagnosed as neuropathy, later unsuccessfully treated as ischaemic heart disease though severe stenosis of the diagonal branch of left anterior descending artery was confirmed by computer tomography angiography. He was finally diagnosed as CP after carefully reading the initial computed tomography. The gross pathology of heart *in situ* originally observed at the time of pericardectomy indicated fibrinous pericarditis, massive haemorrhagic pericardial effusion (300 mL), and thickened pericardium (maximum thickness more than 6 mm). Following pericardial tissue biopsy, the histopathology showed chronic fibrinous pericarditis, without a clear aetiology. His symptoms gradually disappeared after surgical pericardectomy. At the 1-year follow-up visit, the patient complained of no discomfort.

**Discussion:**

Constrictive pericarditis is one of the serious diseases commonly misdiagnosed. Computed tomography and echocardiography show the important diagnostic role in patients with CP, and surgical pericardectomy shows the potential in treating this disease, in some of which the mechanism underlying large haemorrhagic pericardial effusion remains unclear.

**Learning points**

- Constrictive pericarditis (CP) has a long symptom-free period that may take weeks to decades to develop and it is often a clinical diagnosis made with routine investigations. The symptoms and signs of CP are sometimes ignored, leading to a misdiagnosis or delayed diagnosis.
- Echocardiography has an important diagnostic role in patients with CP. Surgical pericardectomy can improve the symptoms and quality of life of patients with CP.

## Introduction

The symptoms and signs of constrictive pericarditis (CP) are often elusive at onset, with a long symptom-free period that may take weeks to decades to develop after an episode of CP or pericardial injury, leading to a misdiagnosis or a delayed diagnosis.^1–3^ CP can be secondary to any pericardial disease and, its course is affected by various diseases.^4^ Data from developed countries indicate that idiopathic and viral pericarditis account for 42–49% of CP cases, and infection (tuberculous or purulent pericarditis) accounts for 3–6% of CP cases.^5^ Tuberculosis (TB) is a primary aetiological factor of CP in developing countries.^6^ The diagnosis of CP is based on the clinical manifestations, imaging, and cardiac catheterization.^4^ Here, we present a case of a patient initially complaining of lower extremity fatigue, intermittent chest tightness, and shortness of breath. He was first misdiagnosed as neuropathy, later unsuccessfully treated as ischaemic heart disease, because the symptoms of chest tightness and shortness of breath were thought to be partially due to severe stenosis of the diagonal branch of left anterior descending artery, which was confirmed by computed tomography angiography (CTA). He was diagnosed as CP after carefully reading the initial computed tomography (CT) scan and finally confirmed by pericardectomy. However, the mechanism underlying large haemorrhagic pericardial effusions remains unclear. His symptoms gradually disappeared after surgical pericardectomy. By reporting this case, we aimed to highlight the important diagnostic role of echocardiography and CT in patients with CP and the potential of surgical pericardectomy in treating CP, promoting the management of this disease.

## Timeline

**Table:** 

Time	Events
0 month	Chief complaint of lower extremity fatigue, severe pitting oedema over both legs and much worse over the right leg, intermittent, and mild chest tightness.
2 months	First misdiagnosed as neuropathy: visited the clinic of neuropathy. The computer tomography, diffusion-weighted imaging, and angiography were unremarkable. The initial transthoracic echocardiogram indicated left ventricular ejection fraction (LVEF) of 72%, fractional shortening (FS) 42%, stroke volume (SV) 88 mL, and cardiac output (CO) 10.9 L/min. Moderate pericardial effusion was detected and the depth of liquid in left ventricular apex was 5 mm and diaphragmatic surface of right ventricle was 12 mm. After the treatment with diuretic, there was little improvement of lower extremity fatigue, oedema of lower extremity, and chest tightness.
4 months	The symptoms of fatigue, chest tightness, and dyspnoea were unsuccessfully treated as ischaemic heart disease: computed tomography angiography showed that the diagonal branch of left anterior descending artery was severely narrow, pleural effusion, mild pericardial effusion, and the thickening pericardium (all evidences of the thickening pericardium were ignored by the radiologist at this time). However, these syndromes were unsuccessfully treated as ischaemic heart disease. The oedema and shortness of breath was significantly improved after thoracentesis. After removing the drainage tube due to the concerns of iatrogenic infection and discomfort; however, the bilateral pleural effusion shortly reappeared.
5 months	Received a diagnosis of constrictive pericarditis (CP): visited the clinic of pulmonary medicine and complained of cough, expectoration, progressive dyspnoea, and pitting oedema over both legs. After reviewing the initial results, the thickened pericardium in high-resolution CT was observed. Constrictive pericarditis is under consideration though there was no Kussmaul sign. This urged the physicians to get informed consent for pericardectomy.
5 months + 1 week	The gross pathology during pericardectomy indicating fibrinous pericarditis, massive haemorrhagic pericardial effusion, and thickened pericardium. After extensive work excluding malignancy and tuberculosis, the patient finally received a diagnosis of idiopathic CP, though the mechanism underlying it remains unproved.
5 months + 2 weeks	All symptoms disappeared
6 months	The echocardiogram indicated normal left ventricular function with the data of LVEF 76%, FS 44%, SV 75 mL, and CO 7.8 L/min.
12 months	At the most recent follow-up, the patient complained of no discomfort.

## Case presentation

A 58-year-old man was brought to the local hospital with chief complaints of lower extremity fatigue, severe bilateral lower extremity pitting oedema, particularly of the right leg, and intermittent, mild chest tightness. He first visited the neuropathy clinic. The CT, diffusion-weighted imaging, and angiography were unremarkable. Haemoglobin was 108 g/L (normal range within 130–175 g/L), urine protein was 0.43 g/24 h (normal range within 0.00–0.15 g/24 h), and albumin was 34.7 g/L (normal range within 40.0–55.0 g/L). Thyroid function was unremarkable.

The initial transthoracic echocardiogram indicated the internal diameter of the ascending aorta 32 mm, right ventricular outflow 30 mm, the left atrium 30 mm, left ventricular (LV) end − diastolic/systolic dimension 50 mm/29 mm, LV dimension 18 mm, and LV function was normal with an ejection fraction (LVEF) of 72%, fractional shortening (FS) of 42%, stroke volume (SV) of 88 mL, and cardiac output (CO) of 10.9 L/min. A moderate pericardial effusion was detected, and the depth of liquid at the left ventricular apex was 5 mm. The diaphragmatic surface of the right ventricle was 12 mm. After the treatment with diuretic, there was little improvement in lower extremity fatigue, oedema of the lower extremities, or chest tightness.

**Figure 1:**
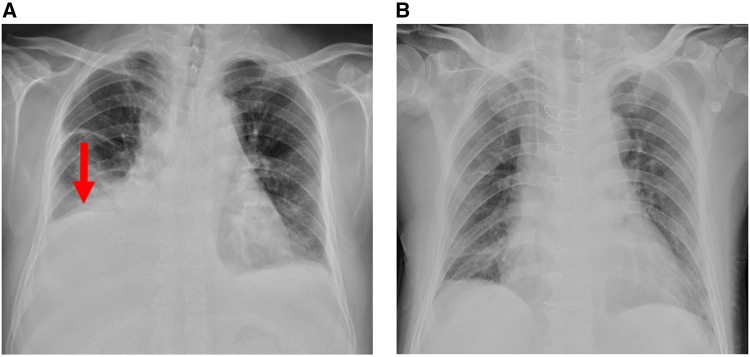
The chest X-ray on admission (*A*) showed high density involving both lungs symmetrically and massive pleural effusion, and chest X-ray (*B*) after pericardectomy indicated significant improvement. Red arrow indicates pleural effusion.

**Figure 2:**
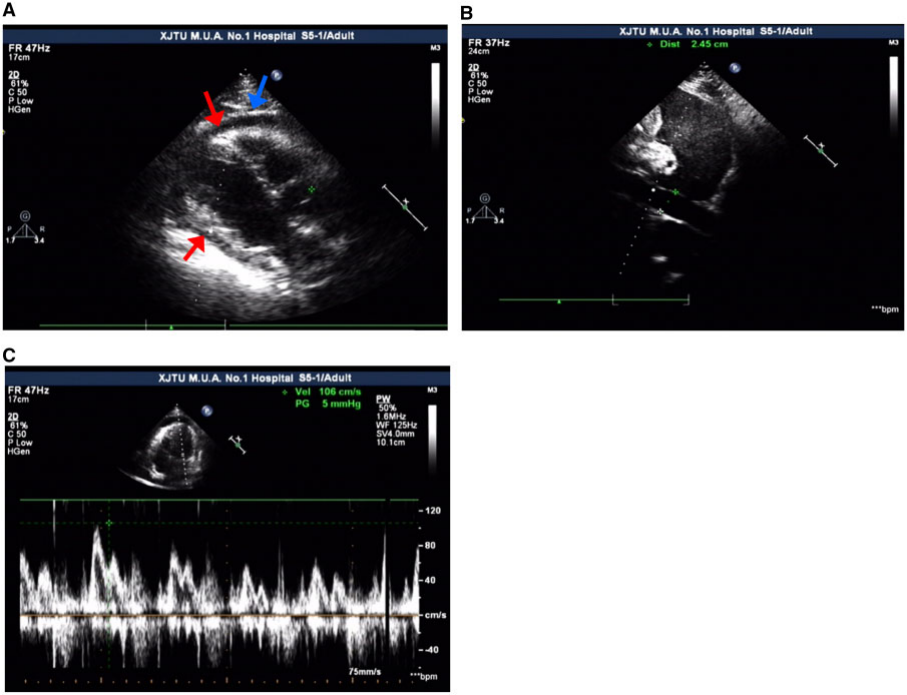
Echocardiogram showed (*A*) small-medium pericardial effusion (red arrows) with the data of left ventricular posterior wall 6.4 mm, right ventricular anterior wall 7.7 mm, apex of heart 6 mm, right ventricular free wall 17 mm, left ventricular free wall 6 mm, and thickened parietal pericardium (blue arrow); however, which was not observed by the sonographer. (*B*) Dilation of the inferior vena cava (24 mm). (*C*) Respiratory variation of the mitral peak E velocity of >25%.

**Figure 3:**
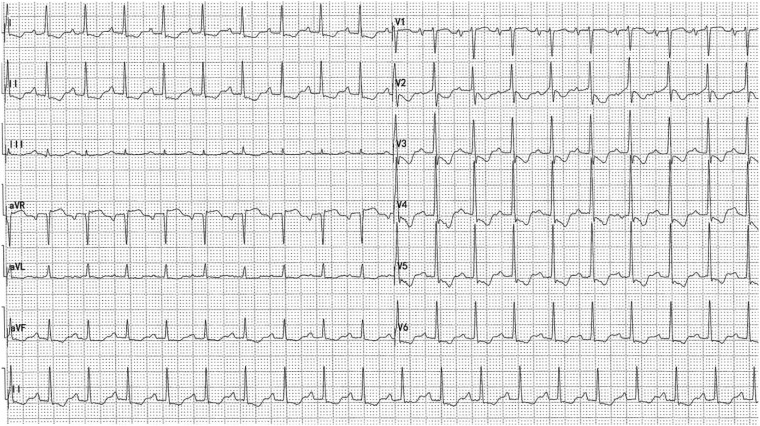
Electrocardiography showed sinus tachycardia and T wave inversion (25 mm/s, 10 mm/mv).

**Figure 4:**
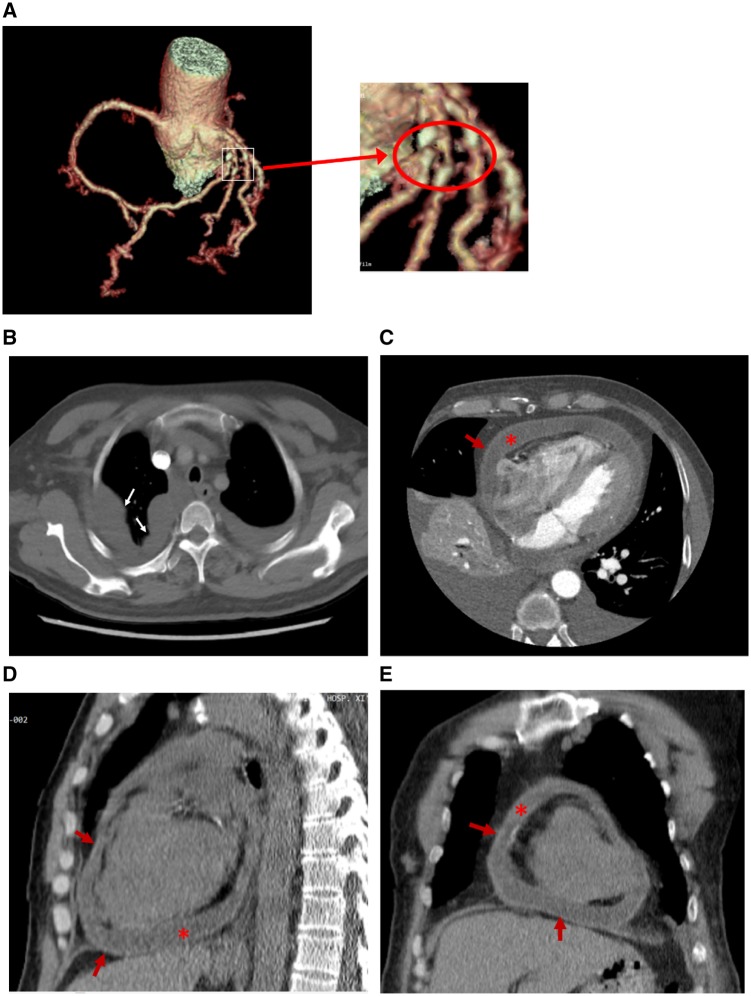
Computed tomography angiography scan on admission showed severe stenosis of the diagonal branch of left anterior descending artery (*A*), pleural effusion (*B*), mild pericardial effusion and the thickening pericardium (*C–E*). However, all evidences of the thickening pericardium were ignored by the radiologist. White arrows indicate irregular fluid levels and encapsulated effusion, supporting the chronic process. Red arrows indicate thickening pericardium. The pericardial thickness by high-resolution computed tomography was more than 4.5 mm. Red asterisks indicate pericardial effusion.

Therefore, the patient visited the outpatient clinic of cardiology for further treatment after new facial and ankle oedema appeared and his shortness of breath persisted. On admission to the cardiology department, a chest X-ray revealed bilateral pleural effusions (*Figure**1A*). Echocardiogram showed normal left ventricular function with an LVEF of 65%, FS of 35%, SV of 76 mL, CO of 9.2 L/min, a small-medium pericardial effusion (left ventricular posterior wall 6.4 mm, right ventricular anterior wall 7.7 mm, apex of the heart 6 mm, right ventricular free wall 17 mm, left ventricular free wall 6 mm), increased size of the right heart (right atrial diameter 36 mm × 46 mm, right ventricle internal dimension 40 mm × 70 mm), dilation of the inferior vena cava (24 mm) and respiratory variation of the mitral peak E velocity of >25% (*Figure**2*). Electrocardiography showed sinus tachycardia, and T wave inversion (*Figure**3*). The coronary CTA and computed tomography pulmonary angiogram (CTPA) showed severe narrowing of the diagonal branch of left anterior descending artery (*Figure**4**A*), as well as the presence of pleural effusion (*Figure**4**B*) and peritoneal effusion. Thoracentesis of the left thoracic cavity using imaging guidance was performed to determine the cause of the excess pleural effusions and to relieve his shortness of breath. A total of an estimated 1180 mL of fluid was drained from the pleural effusion during hospitalization. Laboratory tests of the pleural effusion indicated leakage fluid with the following results: light yellow turbid liquid, Rivalta test (+), total cells count 152 × 10^6^, monocytes accounting for 90% of total white blood cells, white blood cells count 140 × 10^6^, protein 27.7 g/L, glucose 11.87 mmol/L, lactate dehydrogenase (LDH) 106 U/L, and adenosine deaminase (ADA) 4 U/L. Thoracentesis of the right thoracic cavity was also performed later. A total of 3050 mL of fluid was drained from the pleural effusion during hospitalization. Laboratory tests of the pleural effusion indicated leakage fluid with the following results: light yellow turbid liquid, Rivalta test (+), total cells count 352 × 10^6^, monocytes accounting for 72% of total white blood cells, white blood cell count 287 × 10^6^, protein 31.6 g/L, glucose 12.55 mmol/L, LDH 111 U/L, and ADA 4 U/L. Hyperplastic mesothelial cells and lymphocytes were found in the pleural effusion. The oedema and shortness of breath was significantly improved after thoracentesis. After removing the drainage tube due to the concerns of iatrogenic infection and discomfort, however, the bilateral pleural effusions quickly reappeared. Based on the CTA result indicating the severe stenosis of the diagonal branch of left anterior descending artery, antiplatelet and lipid-lowering medications, and a beta-blocker were prescribed because the symptoms of fatigue, chest tightness, and dyspnoea were partially thought to be due to ischaemic heart disease. However, these symptoms were unsuccessfully treated as ischaemic heart disease.

**Figure 5:**
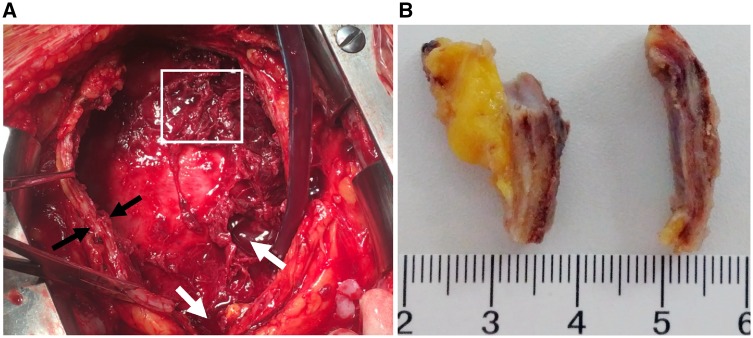
(*A*) The gross view of heart *in situ* originally observed at the time of pericardectomy indicated fibrinous pericarditis, massive haemorrhagic pericardial effusion, and thickened pericardium. (*B*) The maximum thickness of the pericardium is more than 6 mm. Black arrows indicate thickened pericardium, white arrows indicate haemorrhagic pericardial effusion, and white box indicates massive fibrinous pericarditis.

**Figure 6:**
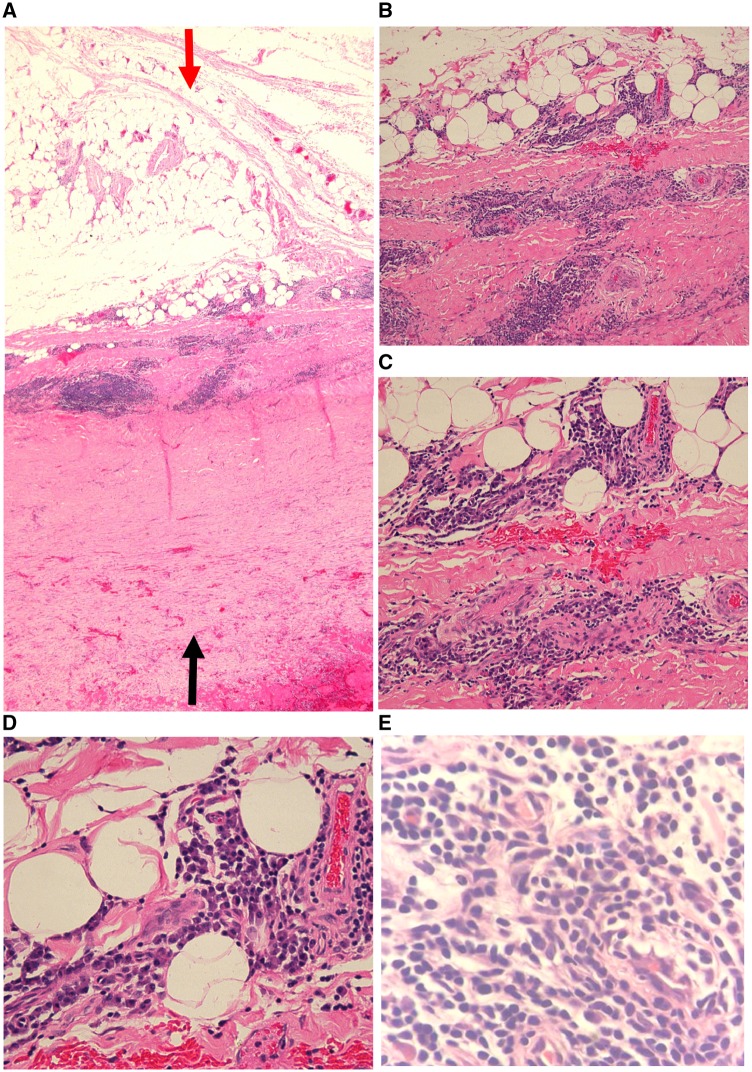
Histopathology, haematoxylin and eosin staining of the pericardial tissue biopsy at different magnification of 4×, 10×, 20×, 40×, and 100×, respectively, shows massive chronic inflammatory cell infiltration (phlogocytes and leucomonocytes) and fibrinoid necrosis (hyaline degeneration). Red and black arrows indicate the outer and interior of cardiac pericardium, respectively.

After being discharged from the department of cardiology without a clear aetiology, the patient complained of cough, expectoration, progressive dyspnoea, and bilateral lower extremity pitting oedema. He then visited the department of pulmonary medicine. After reviewing the initial CT results, a thickened pericardium was observed (*Figure**4**C–E*). Though there was no evident Kussmaul sign, CP was considered and this urged the physicians to get informed consent to perform a pericardectomy. The gross view of the heart *in situ* originally observed during pericardectomy indicated fibrinous pericarditis, a massive haemorrhagic pericardial effusion, and thickened pericardium (*Figure**5**A* and Supplementary material Video S1). The maximum thickness of the pericardium was more than 6 mm (*Figure**5**B*). Haematoxylin and eosin (H&E) staining of the pericardial tissue biopsy obtained from five different regions of the thickened pericardium showed massive chronic inflammatory cell infiltration (phlogocytes and leucomonocytes) and fibroid necrosis (hyaline degeneration). There were no pathological characteristics of TB or malignancy (*Figure 6*). The patient was diagnosed as idiopathic pericarditis. Chronic, non-specific vascular inflammation was proposed to be responsible for the haemorrhagic pericardial effusion. All of the symptoms gradually disappeared 1 week after pericardectomy. Prior to discharge, the repeated X-ray (*Figure 1B*) indicated disappearance of the cavity effusion. At the first scheduled follow-up visit 1 month after pericardectomy, the echocardiogram indicated normal left ventricular function with an EF of 76%, FS of 44%, SV of 75 mL, and CO of 7.8 L/min. At the 2-, 5-, 7-, and 12-month follow-ups, the patient had no complaints of discomfort.

## Discussion

Our case shows the symptoms and signs of right-sided heart failure, including shortness of breath, pleural effusion, ascites, pericardial effusion, and ankle oedema, all of which are non-specific for the diagnosis of CP. The signs and symptoms of predominantly right-sided heart failure were present with shortness of breath, jugular venous distention, oedema, and ascites, which are seen in 24.7, 89.0, 89.0, and 62.0% of the cases of CP.^7^ The percentages of total CP patients without the sign of Kussmaul’s respirations, pulsus paradoxus, and pericardial knock are 21.0, 20.0, and 20.0, respectively.^8^ In our case, decreasing the amount of pleural and pericardial fluid effectively relieve the patient’s shortness of breath but all symptoms reappeared shortly. CP should have been considered.

Pericardial thickness was present in 37% of CP patients evaluated by echocardiography and 72% by CT.^9^ A threshold of pericardial thickness >3–4 mm, respectively yielded a sensitivity and specificity of 83–91% and 100% for the diagnosis of CP.^10^ Increased pericardial thickness of 3 mm was observed in all patients with CP.^11^ In our patient, the pericardial thickness was more than 4.5 mm by high-resolution CT (*Figure**4**C–E*) and more than 6.0 mm by directly pathological observation (*Figure** 5* and Supplementary material Video S1), leading to the diagnosis of CP. However, the presence of thickened pericardium on CT was missed in the initial diagnosis, in part, because the thickened pericardium was not significant on CT (4 mm) and was complicated by the presence of pericardial effusion (*Figure**4**C–E*). It is easy to overlook a slightly thickened pericardium and pericardial effusions when mixed with cellulose exudate and blood components which were proved during pericardectomy.

In patients with CP, the outer layer of the myocardium has decreased motion similar to the pericardium, while the motion of the inner layer of the myocardium is stronger than that of the outer layer myocardium.^12^ The echocardiogram did suggest the CP, although it showed a small-medium pericardial effusion, an increase in right heart size, dilation of the inferior vena cava, and respiratory variation of the mitral peak E velocity of >25% in our patient. The observed signs could also be caused by an existing pericardial effusion. We did not observe septal bounce, or abnormal motion of the myocardium, which could be related to the various clinical manifestations of CP at different stages or the experience of the individual performing the echocardiogram.

The common causes of large haemorrhagic pericardial effusion are TB and malignancy.^13^^,^^14^ In patients those underwent a pericardiocentesis to relieve cardiac tamponade, 64% had a bloody pericardial effusion. The causes of bloody pericardial effusion are iatrogenic disease (31%), malignancy (26%), complications of atherosclerotic heart disease (particularly acute myocardial infarction) (11%), and idiopathic disease (10%).^15^ Some cases of idiopathic pericarditis present with large haemorrhagic pericardial effusions.^16^^,^^17^ In this case, there was no evidence of TB, malignancy, or autoimmune diseases by laboratory tests and pericardial biopsy except for the infiltration of massive, scattered lymphocytes, and fibroid necrosis in the pericardial tissue (*Figure**6*). Taking into account the aforementioned data, we diagnosed this case as idiopathic CP associating with large haemorrhagic pericardial effusion though the mechanism underlying it remains unproved.

## Lead author biography


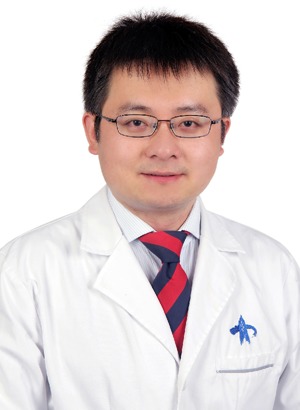


Guoliang Li, MD, PhD, research work focuses on cardiac electrophysiology. He achieved the bachelor degree in medicine and PhD degree from Xi’an Jiaotong University. He studied as a joint training PhD candidate in Institut de Cardiologie of Hôpital de la Salpêtrière in Paris, France. Now, he is a clinical fellow in cardiac electrophysiology in Fuwai hospital in Beijing.

## Supplementary Material

ytz064_Supplementary_Video
