# Supplementary material for: Misdiagnosis of constrictive pericarditis presenting with haemorrhagic pericardial effusion: a case report
Source: Eur Heart J Case Rep. 2019 May 10;3(2):ytz064. doi: 10.1093/ehjcr/ytz064 (PMC6601240; doi:10.1093/ehjcr/ytz064)
Supplement: ytz064_Supplementary_Video [file ytz064_supplementary_video.zip › ytz064-suppl_data/ytz064_Slide_Set.pptx]

## Slide 1
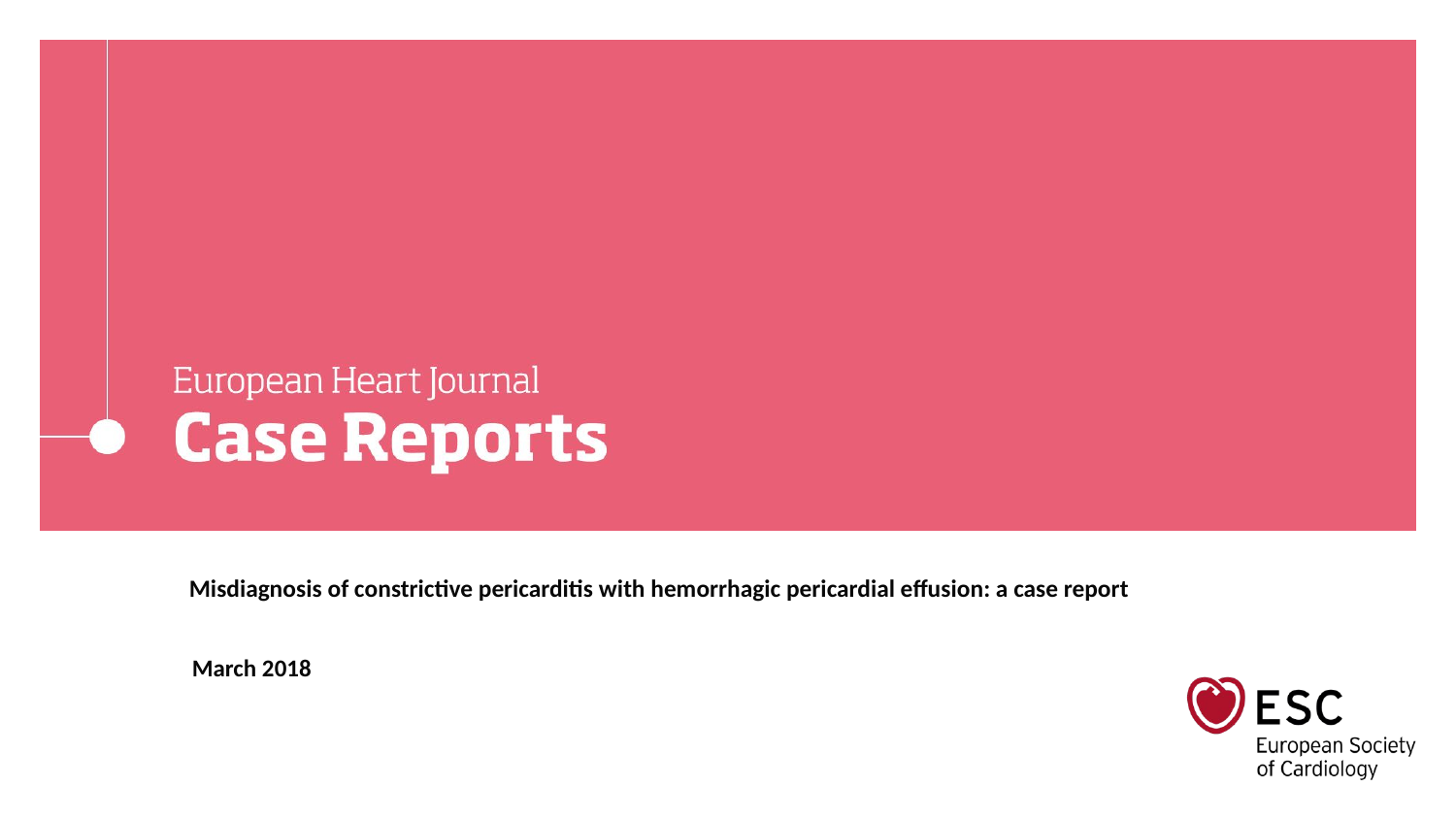

# Misdiagnosis of constrictive pericarditis with hemorrhagic pericardial effusion: a case report
March 2018

## Slide 2
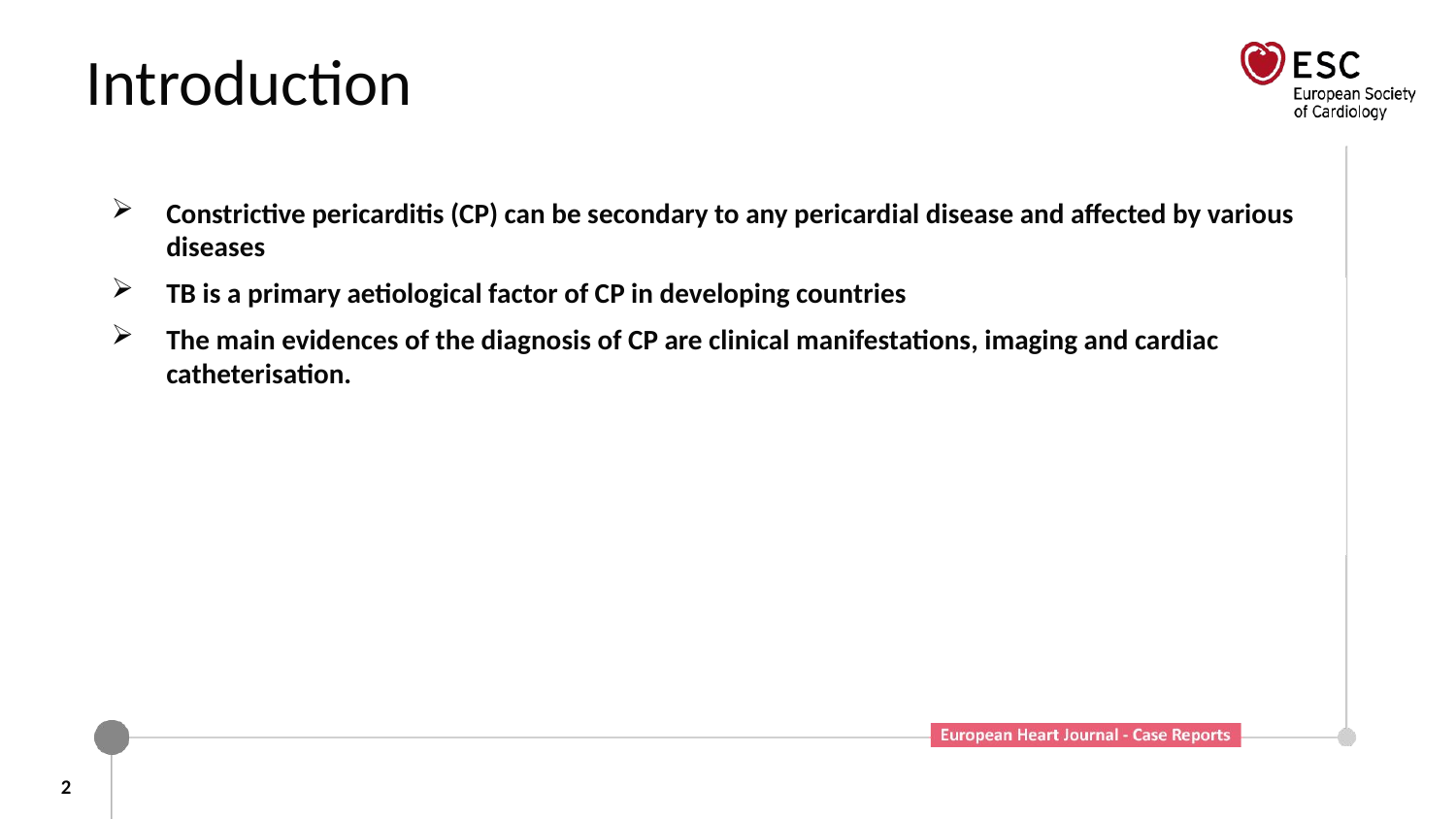

# Introduction
Constrictive pericarditis (CP) can be secondary to any pericardial disease and affected by various diseases
TB is a primary aetiological factor of CP in developing countries
The main evidences of the diagnosis of CP are clinical manifestations, imaging and cardiac catheterisation.
2

## Slide 3
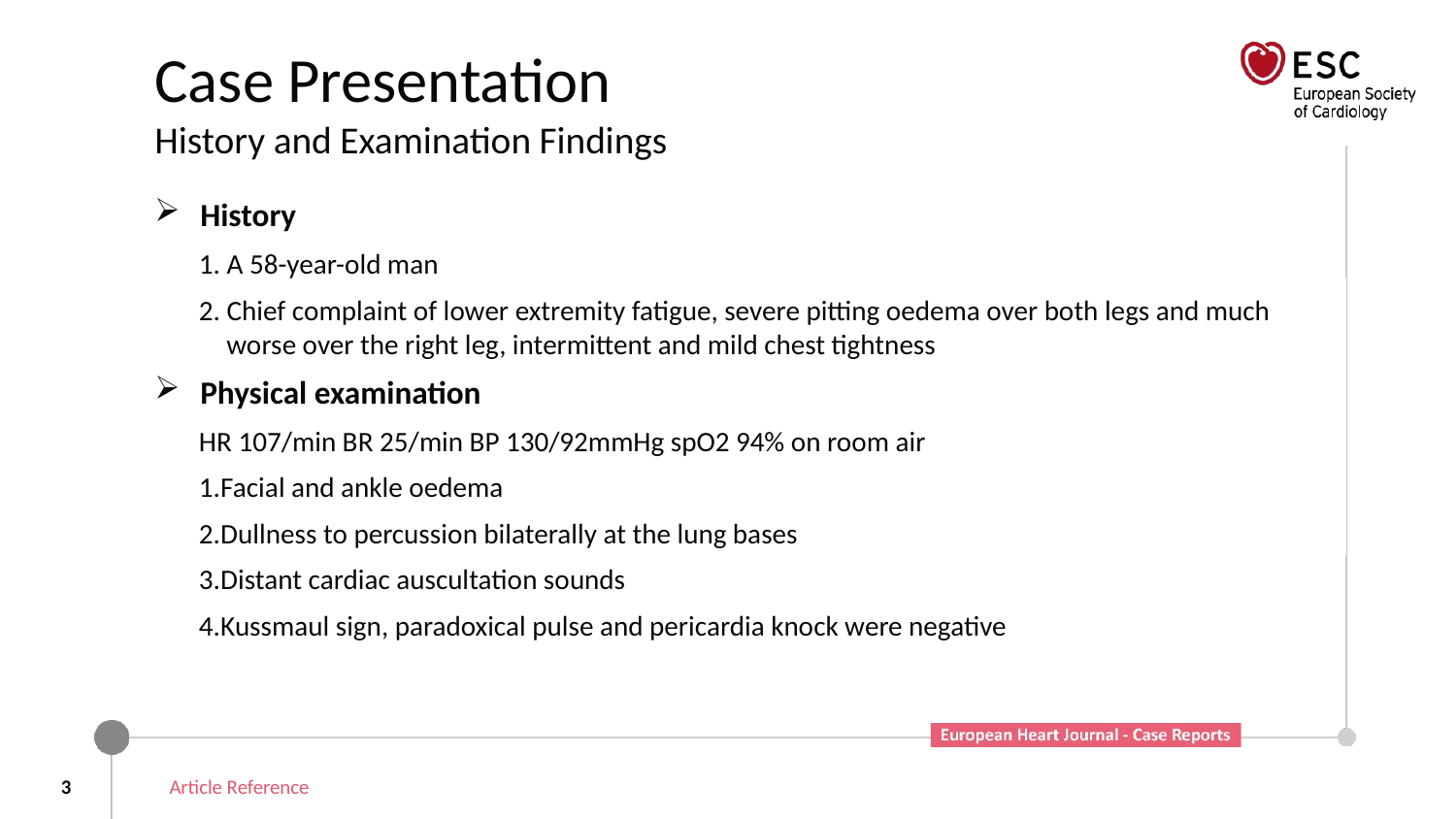

# Case Presentation History and Examination Findings
History
 1. A 58-year-old man
 2. Chief complaint of lower extremity fatigue, severe pitting oedema over both legs and much worse over the right leg, intermittent and mild chest tightness
Physical examination
 HR 107/min BR 25/min BP 130/92mmHg spO2 94% on room air
 1.Facial and ankle oedema
 2.Dullness to percussion bilaterally at the lung bases
 3.Distant cardiac auscultation sounds
 4.Kussmaul sign, paradoxical pulse and pericardia knock were negative
3
Article Reference

## Slide 4
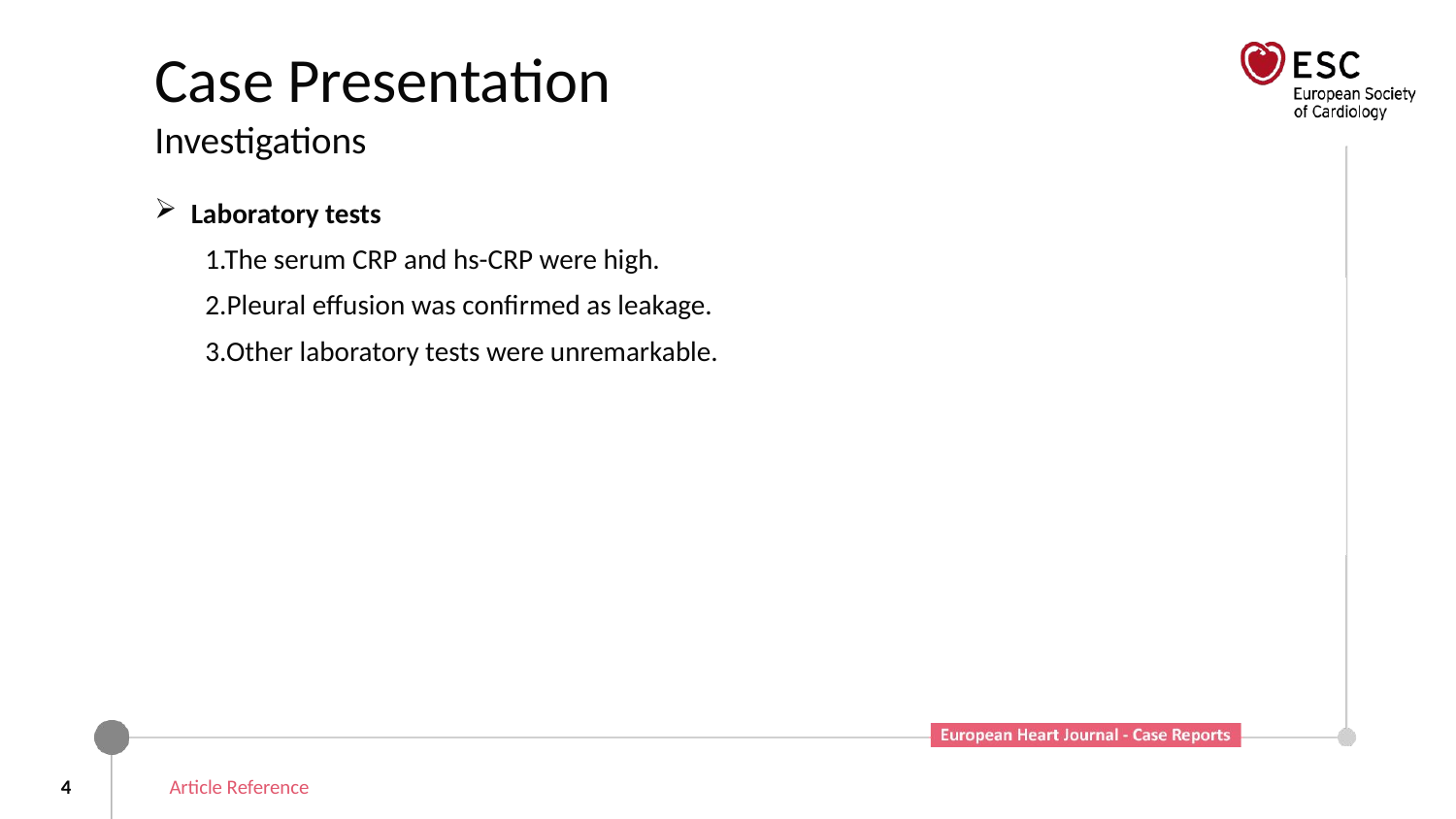

# Case Presentation Investigations
Laboratory tests
 1.The serum CRP and hs-CRP were high.
 2.Pleural effusion was confirmed as leakage.
 3.Other laboratory tests were unremarkable.
4
Article Reference

## Slide 5
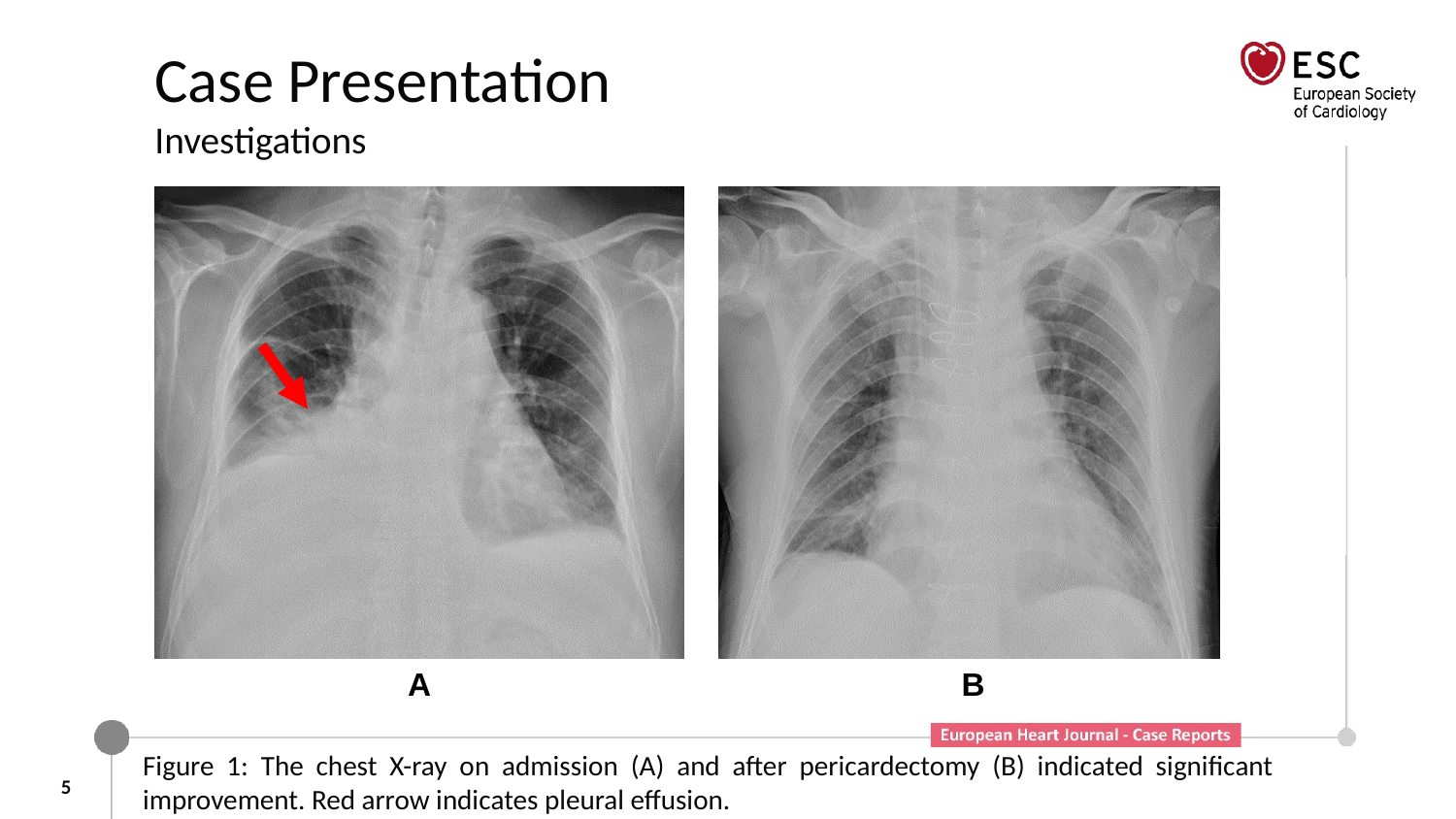

# Case Presentation Investigations
A
B
Figure 1: The chest X-ray on admission (A) and after pericardectomy (B) indicated significant improvement. Red arrow indicates pleural effusion.
5

## Slide 6
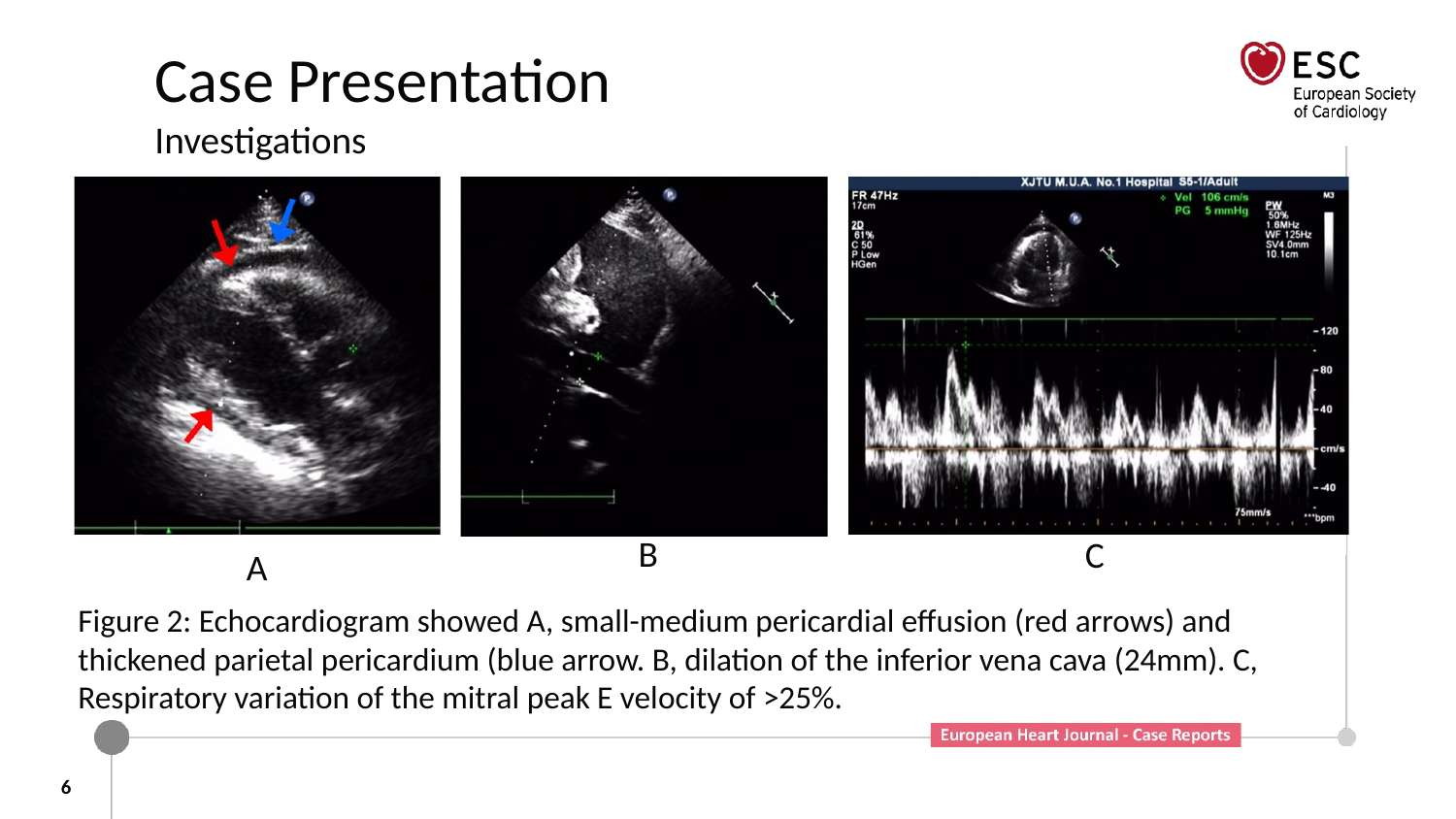

# Case Presentation Investigations
B
C
A
Figure 2: Echocardiogram showed A, small-medium pericardial effusion (red arrows) and thickened parietal pericardium (blue arrow. B, dilation of the inferior vena cava (24mm). C, Respiratory variation of the mitral peak E velocity of >25%.
6

## Slide 7
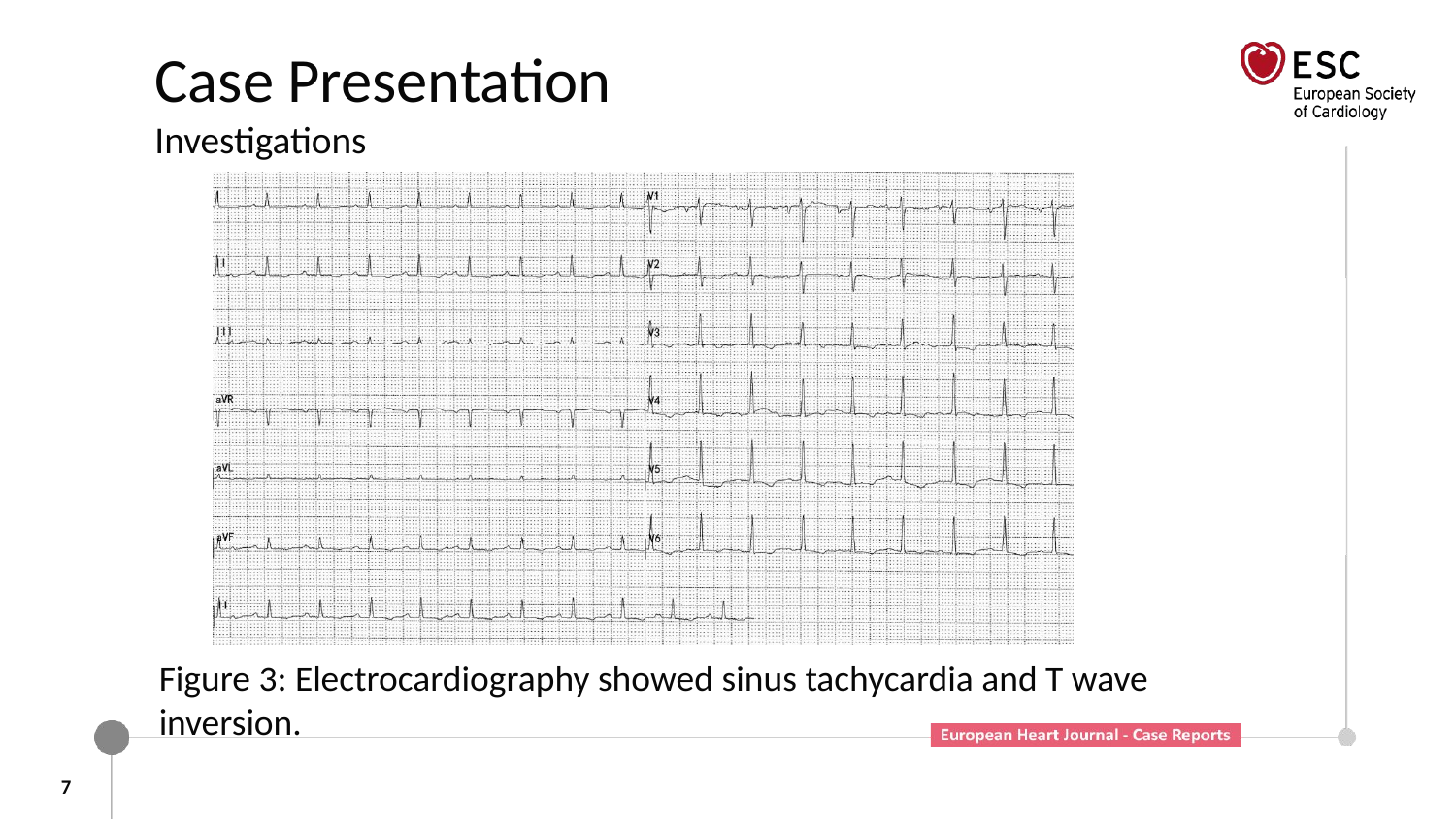

# Case Presentation Investigations
Figure 3: Electrocardiography showed sinus tachycardia and T wave inversion.
7

## Slide 8
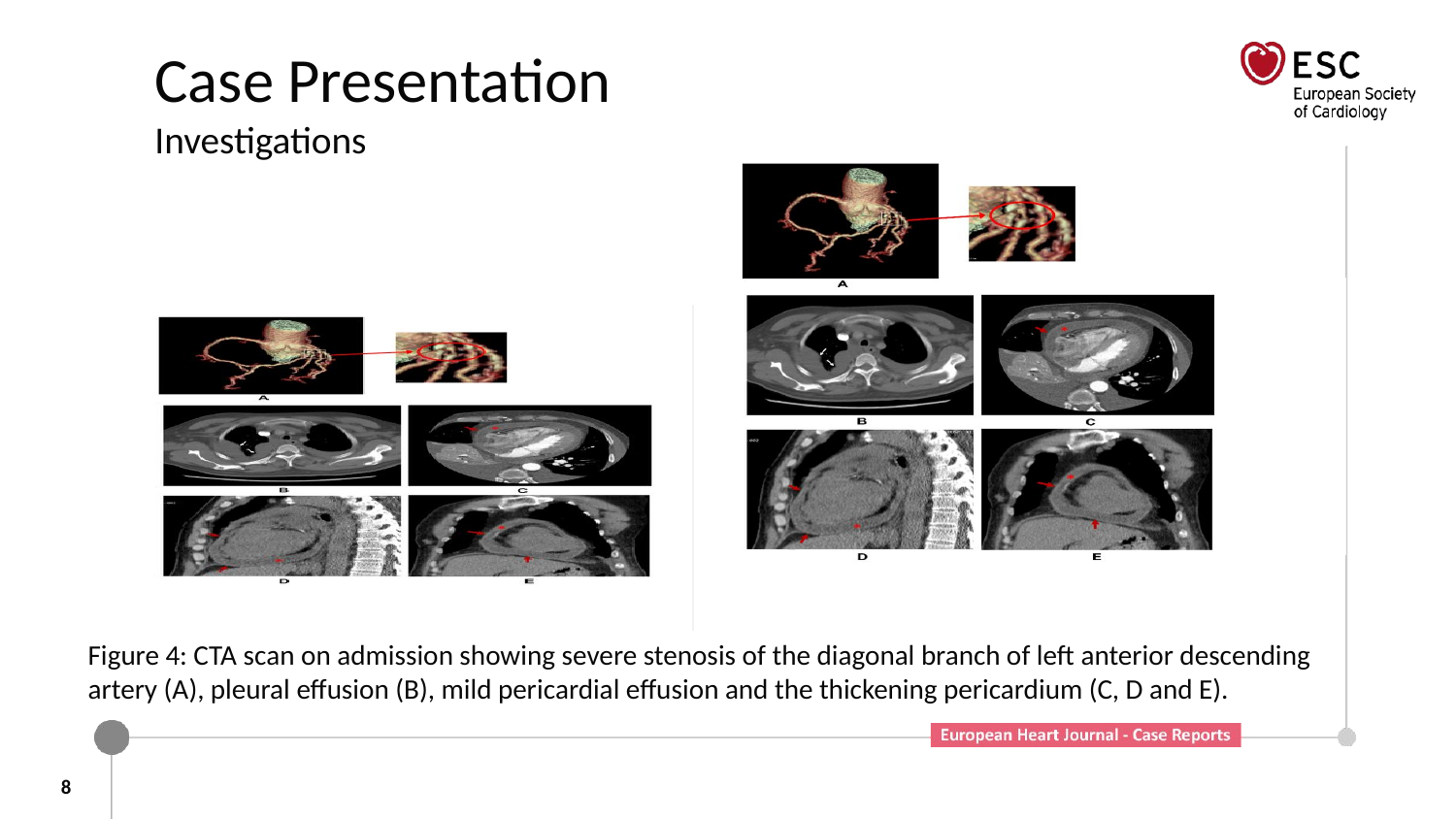

# Case Presentation Investigations
Figure 4: CTA scan on admission showing severe stenosis of the diagonal branch of left anterior descending artery (A), pleural effusion (B), mild pericardial effusion and the thickening pericardium (C, D and E).
8

## Slide 9
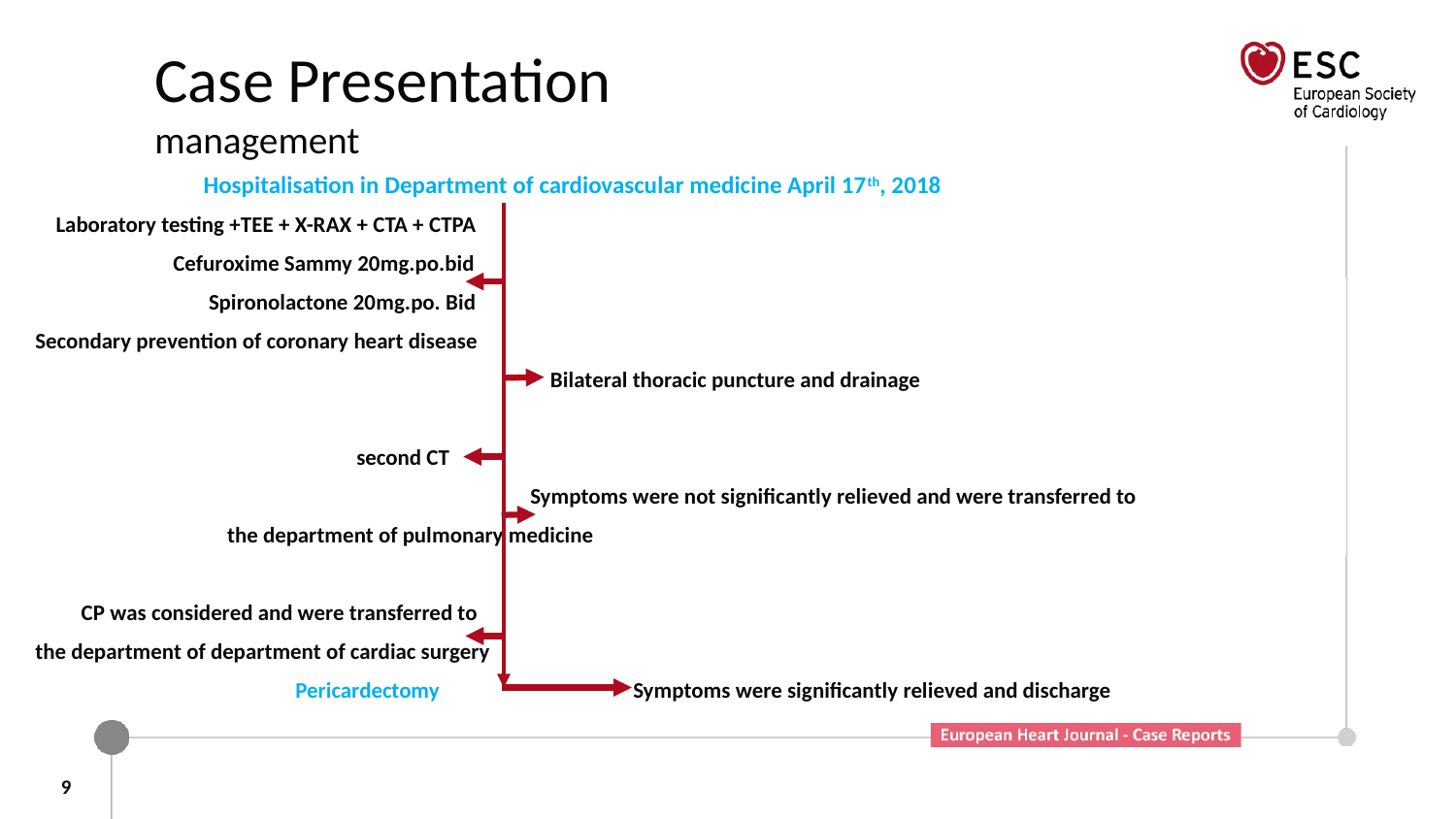

# Case Presentation management
 Hospitalisation in Department of cardiovascular medicine April 17th, 2018
 Laboratory testing +TEE + X-RAX + CTA + CTPA
 Cefuroxime Sammy 20mg.po.bid
 Spironolactone 20mg.po. Bid
 Secondary prevention of coronary heart disease
 Bilateral thoracic puncture and drainage
 second CT
 Symptoms were not significantly relieved and were transferred to
 the department of pulmonary medicine
 CP was considered and were transferred to
 the department of department of cardiac surgery
 Pericardectomy Symptoms were significantly relieved and discharge
9

## Slide 10
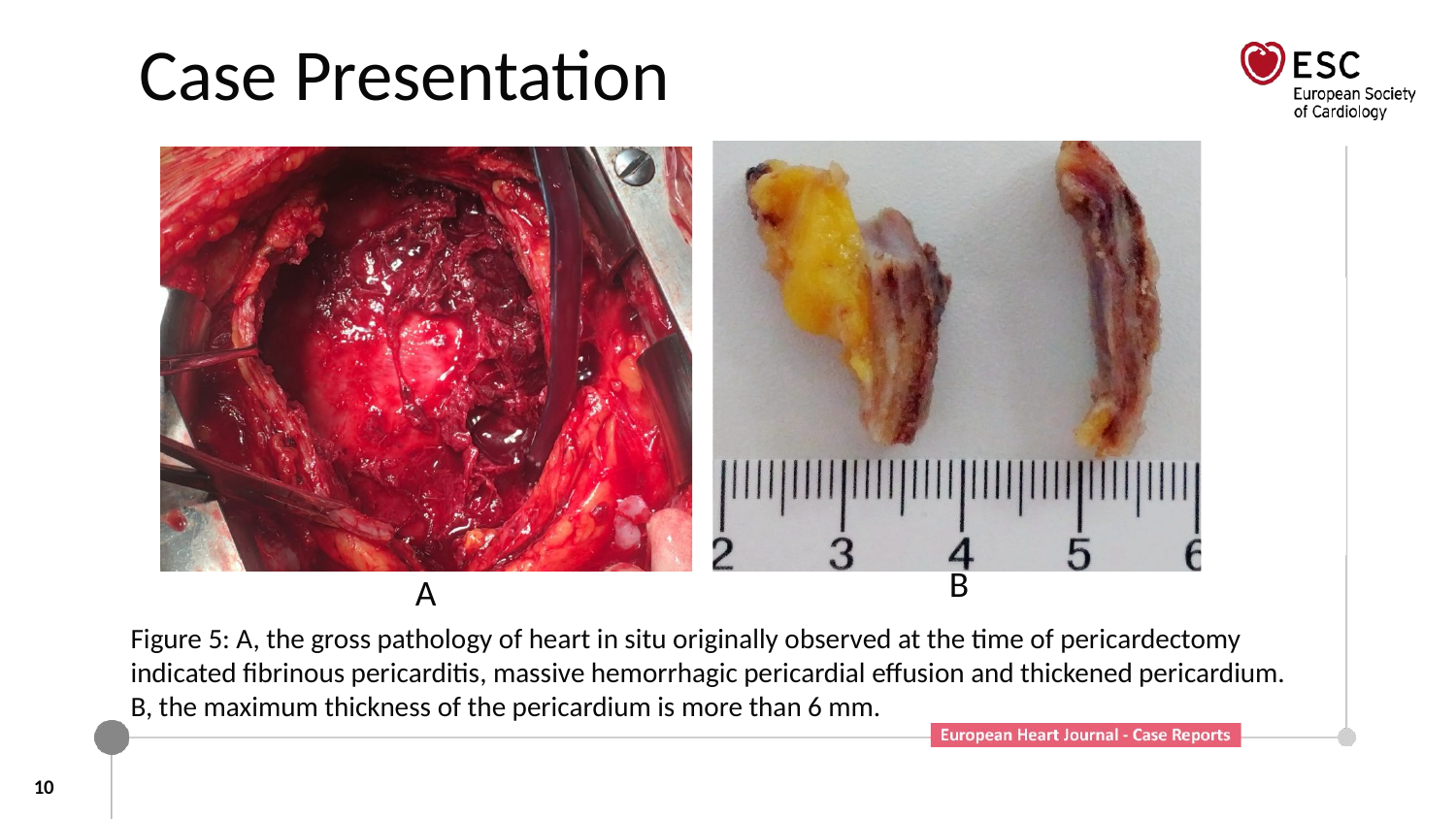

# Case Presentation
B
A
Figure 5: A, the gross pathology of heart in situ originally observed at the time of pericardectomy indicated fibrinous pericarditis, massive hemorrhagic pericardial effusion and thickened pericardium. B, the maximum thickness of the pericardium is more than 6 mm.
10

## Slide 11
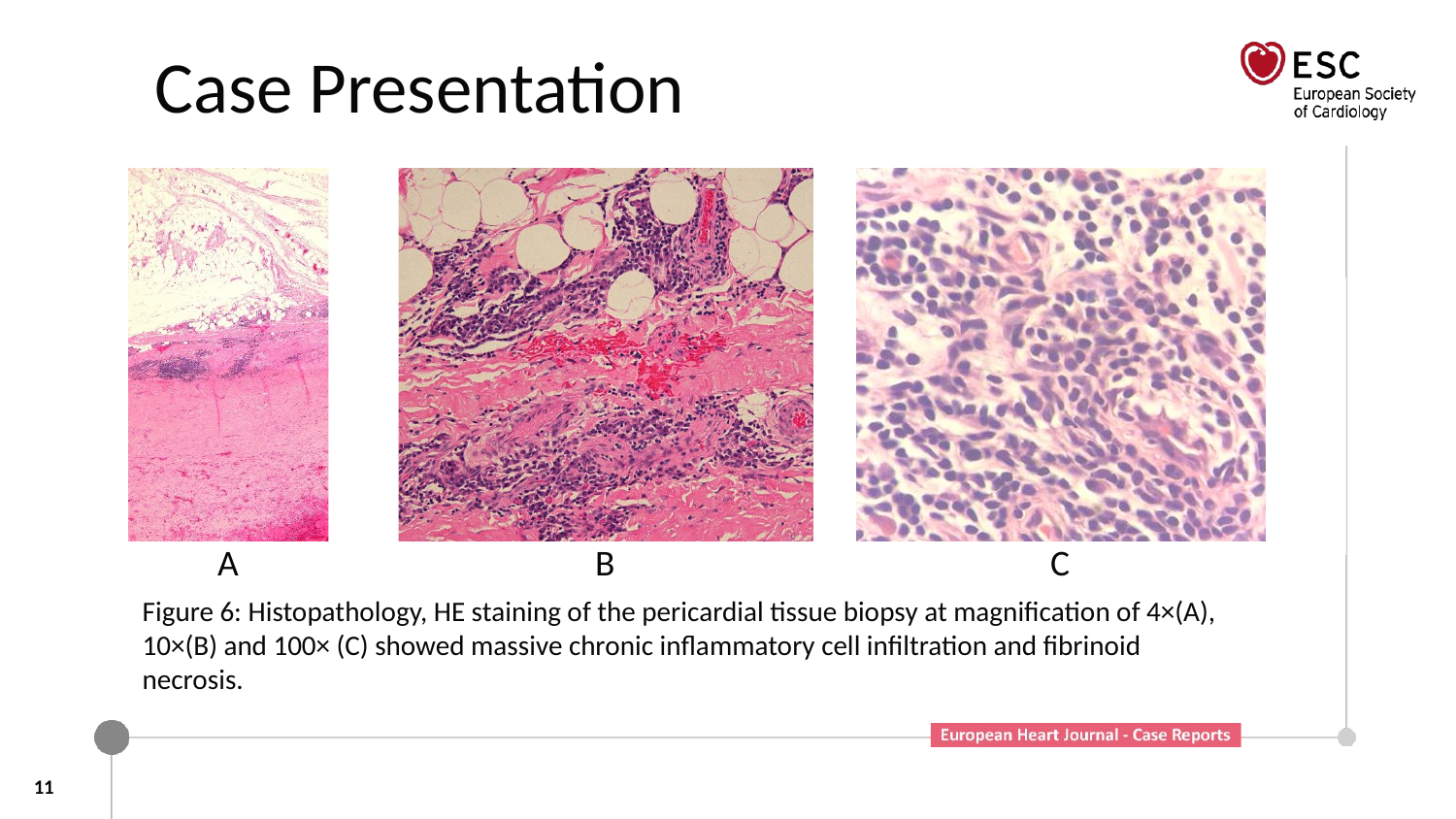

B
# Case Presentation
B
C
A
Figure 6: Histopathology, HE staining of the pericardial tissue biopsy at magnification of 4×(A), 10×(B) and 100× (C) showed massive chronic inflammatory cell infiltration and fibrinoid necrosis.
11
Figure 6: Histopathology, HE staining of the pericardial tissue biopsy at different magnification of 4×, 10×, 20×, 40× and 100×, respectively, showed massive chronic inflammatory cell infiltration (phlogocytes and leukomonocytes) and fibrinoid necrosis (hyaline degeneration)
D
E

## Slide 12
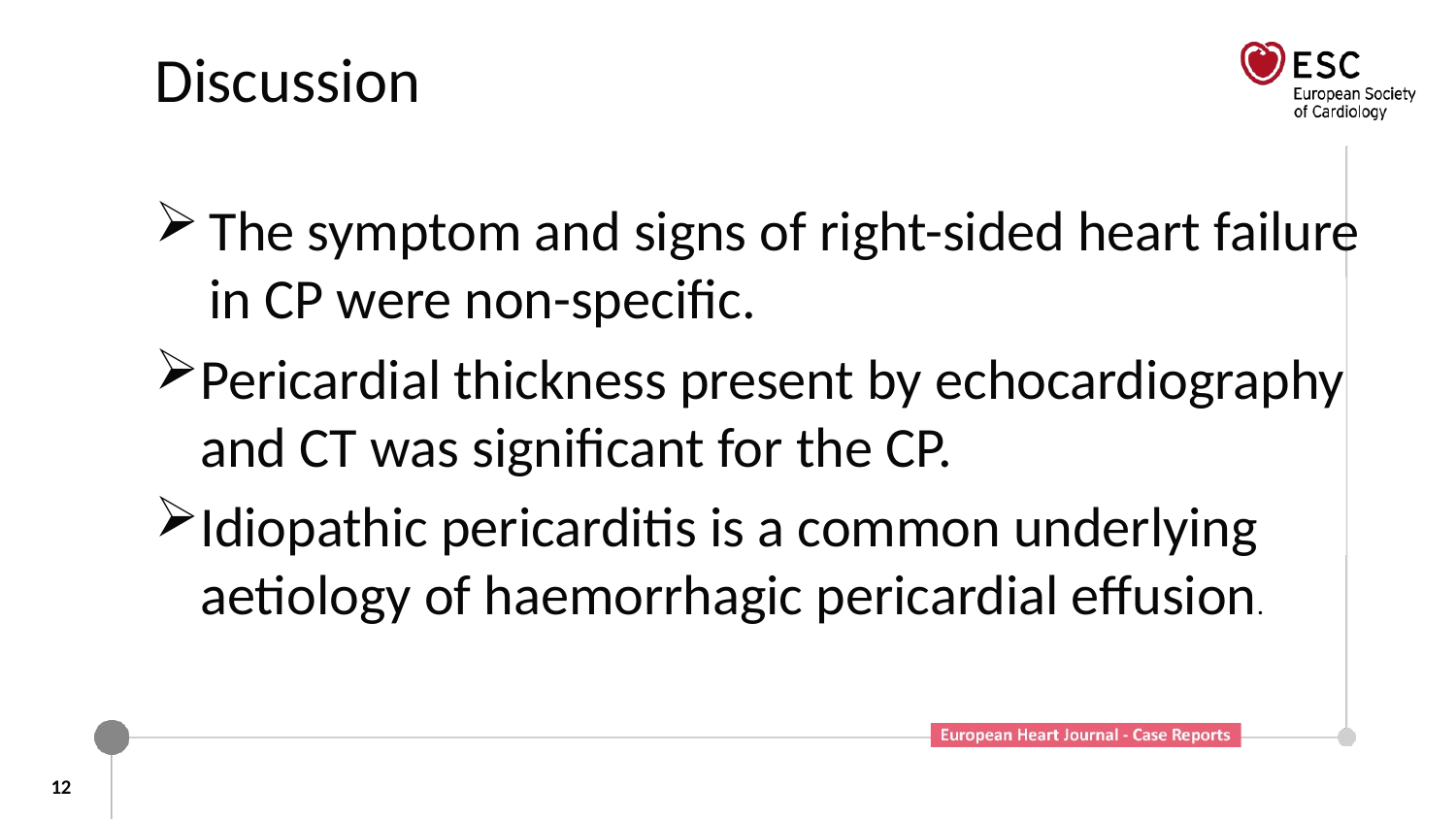

# Discussion
The symptom and signs of right-sided heart failure in CP were non-specific.
Pericardial thickness present by echocardiography and CT was significant for the CP.
Idiopathic pericarditis is a common underlying aetiology of haemorrhagic pericardial effusion.
12

## Slide 13
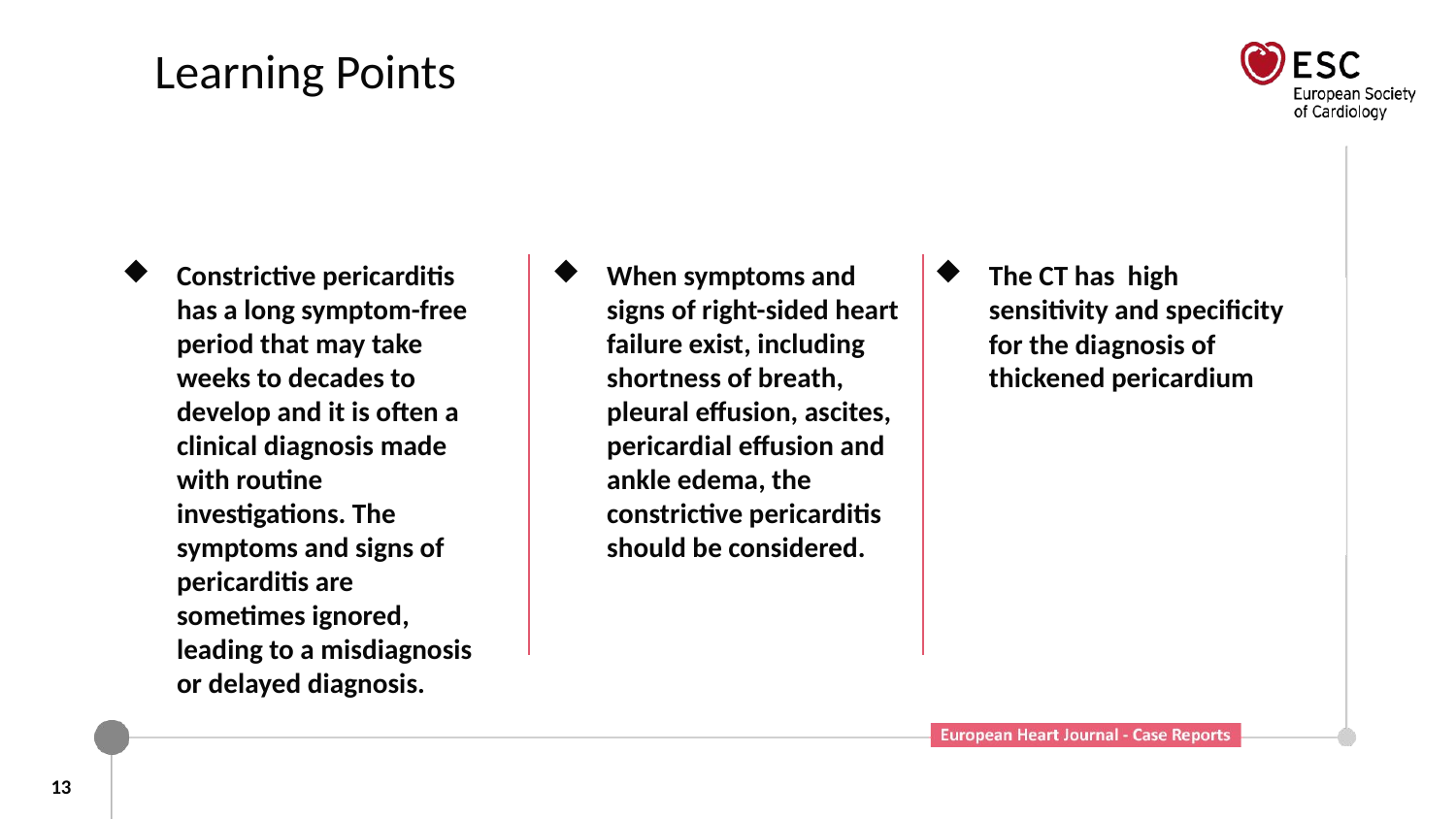

# Learning Points
Constrictive pericarditis has a long symptom-free period that may take weeks to decades to develop and it is often a clinical diagnosis made with routine investigations. The symptoms and signs of pericarditis are sometimes ignored, leading to a misdiagnosis or delayed diagnosis.
When symptoms and signs of right-sided heart failure exist, including shortness of breath, pleural effusion, ascites, pericardial effusion and ankle edema, the constrictive pericarditis should be considered.
The CT has high sensitivity and specificity for the diagnosis of thickened pericardium
13

## Slide 14
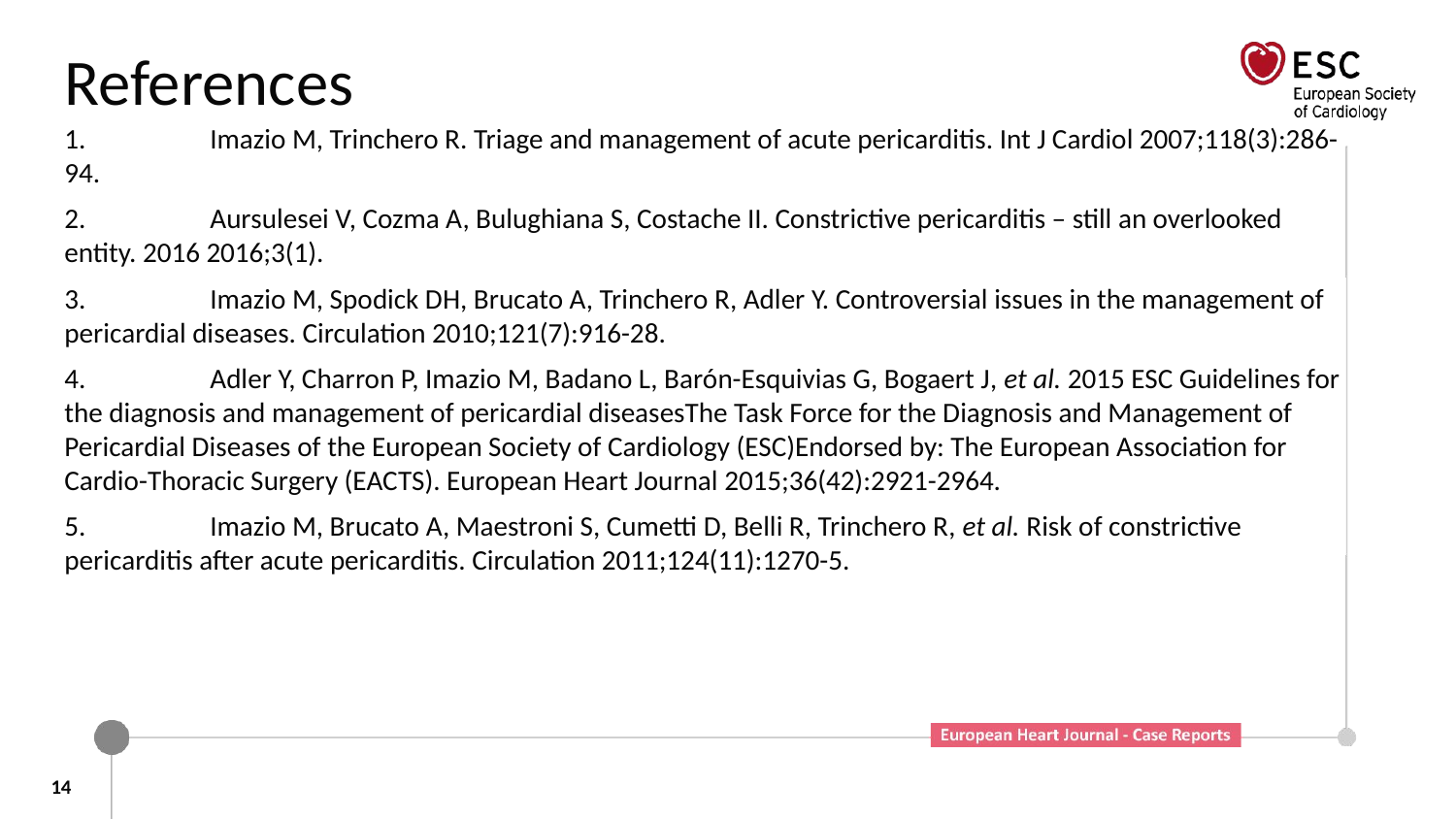

# References
1.	Imazio M, Trinchero R. Triage and management of acute pericarditis. Int J Cardiol 2007;118(3):286-94.
2.	Aursulesei V, Cozma A, Bulughiana S, Costache II. Constrictive pericarditis – still an overlooked entity. 2016 2016;3(1).
3.	Imazio M, Spodick DH, Brucato A, Trinchero R, Adler Y. Controversial issues in the management of pericardial diseases. Circulation 2010;121(7):916-28.
4.	Adler Y, Charron P, Imazio M, Badano L, Barón-Esquivias G, Bogaert J, et al. 2015 ESC Guidelines for the diagnosis and management of pericardial diseasesThe Task Force for the Diagnosis and Management of Pericardial Diseases of the European Society of Cardiology (ESC)Endorsed by: The European Association for Cardio-Thoracic Surgery (EACTS). European Heart Journal 2015;36(42):2921-2964.
5.	Imazio M, Brucato A, Maestroni S, Cumetti D, Belli R, Trinchero R, et al. Risk of constrictive pericarditis after acute pericarditis. Circulation 2011;124(11):1270-5.
14
